# Long-chain noncoding RNA sequencing analysis reveals the molecular profiles of chemically induced mammary epithelial cells

**DOI:** 10.3389/fgene.2023.1189487

**Published:** 2023-09-05

**Authors:** Mengzhen Wei, Wenkui Tang, Danwei Lv, Mingxing Liu, Guodong Wang, Quanhui Liu, Liangshan Qin, Ben Huang, Dandan Zhang

**Affiliations:** Guangxi Key Laboratory of Eye Health, Guangxi Academy of Medical Sciences, People’s Hospital of Guangxi Zhuang Autonomous Region, Nanning, China

**Keywords:** long-chain noncoding RNA (lncRNA), reprogramming, mammary epithelial cells, goat, lactation

## Abstract

Long noncoding RNAs (lncRNAs) were important regulators affecting the cellular reprogramming process. Previous studies from our group have demonstrated that small molecule compounds can induce goat ear fibroblasts to reprogram into mammary epithelial cells with lactation function. In this study, we used lncRNA-Sequencing (lncRNA-seq) to analyze the lncRNA expression profile of cells before and after reprogramming (CK vs. 5i8 d). The results showed that a total of 3,970 candidate differential lncRNAs were detected, 1,170 annotated and 2,800 new lncRNAs. Compared to 0 d cells, 738 lncRNAs were significantly upregulated and 550 were significantly downregulated in 8 d cells. Heat maps of lncrnas and target genes with significant differences showed that the fate of cell lineages changed. Functional enrichment analysis revealed that these differently expressed (DE) lncRNAs target genes were mainly involved in signaling pathways related to reprogramming and mammary gland development, such as the Wnt signaling pathway, PI3K-Akt signaling pathway, arginine and proline metabolism, ECM-receptor interaction, and MAPK signaling pathway. The accuracy of sequencing was verified by real-time fluorescence quantification (RT-qPCR) of lncRNAs and key candidate genes, and it was also demonstrated that the phenotype and genes of the cells were changed. Therefore, this study offers a foundation for explaining the molecular mechanisms of lncRNAs in chemically induced mammary epithelial cells.

## 1 Introduction

### 1.1 Background

LncRNA is an RNA molecule greater than 200 nucleotides in length that does not have coding potential (Okazaki et al., 2002). Based on the location of lncRNAs on the genome relative to protein-coding genes, lncRNAs were broadly classified as positive, antisense, intragenic, intergenic, bidirectional, and overlapping lncRNAs (Kopp and Mendell, 2018). LncRNAs were initially considered to be genomic transcriptional “noise,” a byproduct of RNA polymerase II transcription. Compared with mRNAs, lncRNAs do not have typical start codons, promoter conserved regions, stop codons or open reading frames (Ulitsky et al., 2011; Guttman and Rinn, 2012). However, in recent years, an increasing number of lncRNAs with biological functions have been identified, and lncRNAs play important roles in many biological activities, such as participating in cell proliferation (Liu et al., 2018; Qin et al., 2018), differentiation (Song et al., 2016; Touat Todeschini et al., 2017) and apoptosis (Liu et al., 2017; Nan et al., 2017) and promoting myogenic cell differentiation and injury-induced muscle regeneration (Wang et al., 2015), fat deposition (Wang et al., 2017), lactation (Yu et al., 2017), reproduction (Wang et al., 2014) and immunity (Zhou et al., 2017). lncRNAs have also become a hot spot for research in different scientific directions. LncRNAs are important regulatory molecules of gene expression and have highly diverse biological functions. Their modes of action are broadly divided into three categories: one is at the chromatin level, which regulates gene expression through epistatic modification of chromatin; the second is at the transcriptional level, which regulates gene expression by altering the functions of factors and enzymes that mediate the regulation of gene expression; and the third is at the post transcriptional level, which regulates mRNA by binding to coding genes or miRNAs, thereby regulating the expression levels of mRNAs and miRNAs (Lu et al., 2017).

Studies in humans and model animals have shown that lncRNAs were involved in mammary gland development and the regulation of lactation in mammals (Standaert et al., 2014; Yu et al., 2017). Zhang et al. (Zhang et al., 2014) found that overexpression of lncRNAROR increased the self-renewal of mammary stem cells, and further studies of its function showed that lncRNAROR plays a key role in maintaining a normal stem cell subpopulation in mammary epithelial cells. These findings provide a basis for the biological function of lincRNAs in regulating mammary gland development and susceptibility to mastitis in dairy cows (Tong et al., 2017). It has been found that there are specific lncRNAs in the reprogramming process and in mouse and human embryonic stem cells, and these lncRNAs were strongly correlated with the expression of the important reprogramming factors OCT4, NANOG, and SOX2 (Dinger et al., 2008). LncRNAs affect the cell reprogramming process, and lncRNAROR is one of these regulators, and inhibition of their expression decreases the efficiency of cellular reprogramming (Loewer et al., 2010; Guttman et al., 2011)^.^ However, less is known about goat lncRNAs, and the regulatory relationship between cell reprogramming and mammary gland development and lncRNA expression is unclear.

### 1.2 Objective

The aim of this study was to analyze the expression profile of lncRNAs after small molecule compound-induced reprogramming of goat fibroblasts into mammary epithelial cells, to explore the relationship between lncRNAs and mammary gland development and cell reprogramming regulation in goats and to provide a molecular mechanism to explain the role of lncRNAs in mammary gland development and cell reprogramming regulation in goats on a theoretical basis.

## 2 Materials and methods

### 2.1 Sample collection and RNA extraction

#### 2.1.1 Sample collection

Goat ear margin fibroblasts were extracted and purified by isolation and pre-digestion of ear tissue from 2 to 3 months old Guanzhong dairy goats, provided by the Guangxi Institute of Animal Science. The test samples used were obtained from laboratory-induced goat fibroblasts. Goat mammary epithelial cells were obtained after 8 days of induction of goat fibroblasts using the 5i system (Induction medium composed of Forskolin, TTNPB, VPA, Repsox, Tranyl-cypromine, five small molecule compounds) (Zhang et al., 2021), and cell precipitates were collected from goat fibroblasts (0 day cells, CK group) and induced goat mammary epithelial cells (8 day cells, 5i8d group), with two biological replicates from each group. Samples were collected and stored at −80°C.

#### 2.1.2 RNA extraction and detection

Total RNA was extracted from goat fibroblasts and goat mammary epithelial cells using the TRIzol method. One percent agarose electrophoresis was used to detect the presence of degradation and impurities in the RNA samples, and the purity of the samples was measured by a Kaio K5500 spectrophotometer. The integrity and purity of the RNA samples were measured by an Agilent 2100 RNA Nano 6000 Assay Kit (Agilent Technologies, CA, United States) to detect the integrity and concentration of the RNA samples.

### 2.2 Library construction, sequencing and transcript assembly

Goat fibroblasts and induced goat mammary epithelial cells were each sampled with 3 μg of total RNA as a starting amount to construct the lncRNA libraries. Ribo-Zero™ GoldKits were used to remove rRNA from the samples, and different index tags were selected for library construction according to the operating instructions of the NEB Next Ultra Directional RNA LibraryPrep Kit for Illumina (NEB, Ipswich, United States).

The specific steps of library construction were as follows: first, a kit was used to remove ribosomal rRNA, fragmentation buffer was added to the reaction system to fragment the RNA into short fragments, the fragmented RNA was used as a template to synthesize the first strand of cDNA with six-base random primers (random hexamers), and buffer, dNTPs, RNase H and DNA polymerase I were added to synthesize the second strand of cDNA. The cDNA second strand was purified by a QiaQuick PCR kit and eluted with EB buffer, end-repair, base A, sequencing junction, agarose gel electrophoresis to recover the target size fragment, digestion of cDNA second strand with UNG enzyme, PCR amplification, and finally agarose gel electrophoresis to recover the target size fragment. The whole library preparation was completed by agarose gel electrophoresis. Finally, the constructed libraries were used for Illumina sequencing.

### 2.3 Screening of differentially expressed lncRNAs and target gene prediction

LncRNAs were classified into intergenic RNAs (lincRNAs), introinc lncRNAs, anti-sense lncRNAs, sense lncRNAs, bidirectional lncRNAs and other types according to their position in relation to the coding sequence. Among them, the proportion of lincRNAs was the highest, and the screening of novel lncRNAs was mainly for lincRNAs, intronic lncRNAs and antisense lncRNAs. A series of screening conditions were set, and the resulting lncRNAs were used as the final candidate novel lncRNAs for subsequent analysis. The basic screening conditions were as follows: the transcript length was greater than or equal to 200 bp, the number of exons was greater than or equal to 2, the read coverage of each transcript was calculated, transcripts with less than 5 in all samples were screened out, and the known mRNAs and other noncoding RNAs (rRNA, tRNA, snoRNA, snRNA, etc.) in the species were screened out by comparison with the annotation file of the species using gffcompare. Whether the new transcripts have coding potential is the key condition to determine whether the new transcripts were lncRNAs. By using the lncRNA information from the previous step of the initial screening, a variety of coding potential analysis software programs were integrated for screening, mainly CNCI analysis, CPC analysis, PFAM protein structural domain analysis, and CPAT analysis (animal only). Several analysis methods discriminated noncoding transcripts as the final novel lncRNA data set. Differential expression analysis was performed from candidate lncRNAs using DEseq to compare the treatment group with the reference group, and genes with |log2Ratio|≥2 and *p* < 0.05 were selected as significant differential expression screening conditions to obtain the number of up and downregulated genes. For differentially expressed lncRNA target genes, cis and trans target analyses were performed to indirectly predict their functions by target genes.

### 2.4 GO (Gene ontology) enrichment and KEGG (Kyoto encyclopedia of genes and genomes) pathway analysis

DAVID was used online for GO analysis and KEGG pathway analysis of the predicted target genes (Sherman et al., 2022).

### 2.5 RT-qPCR validation of lncRNA and target genes

Six lncRNAs were randomly selected and subjected to RT-qPCR. U6 was selected as the internal reference gene, and the primers were all synthesized by Janus Biological Engineering Co. The total fluorescence PCR system was 20 μL: 0.5 μL of each upstream and downstream primer (20 μmol/L), 1 μL of cDNA, 8 μL of ddH2O, and 10 μL of 2X lncRNA PreMix. The PCR conditions were as follows: 95°C for 3 min; 40 cycles of 95°C for 5 s and 60°C for 15 s; and 3 replicates. The relative expression of lncRNAs in induced goat mammary epithelial cells was calculated by the 2^−ΔΔCT^ method and verified by comparison with the transcriptome sequencing results. The target gene qRT-PCR method was consistent. The lncRNA and target gene primer sequence informations were shown in Table 1.

**TABLE 1:** lncRNA primer sequence information.

lncRNAs	Primer sequence (5′-3′)	Product size/bp
MSTRG12675	F:AGGCAAAGAACAGTCAGGCA	136
	R:TGCTGGTAATTGAGGGTCGG	
LOC102172108	F:AGGTGTGTGTTACTGCGAGG	113
	R:CTTCCACTAACTTGCCGGGT	
LOC106503513	F:GACCTTGACTGTGAAGCGGA	121
	R:TTCCTCAAATCACCGGGGTG	
MSTRG25656	F:CATGGCAAGCCGCTATTGAC	127
	R:AGAACCCAGCCACCATTCTG	
MSTRG29368	F:CCTGAGGCCTCCGTGAAAAT	127
	R:GAAACTCTGTGCCGGACTGA	
MSTRG15634	F:GAGACCCAGTGCAACCAAGA	140
	R:TGCCCTCTGCCCTGAATTTT	
U6	F:CGCACAGACATACGTCCCC	156
	R:TGGTCGGCAGTAAAGCAGAAT	
FGF9	F:AACTGGTACAACACGTACTCC	123
	R:TTTCTGGTGCCGCTTAGTCC	
PDGFRA	F:TCACGGAGATCACCACTGACA	86
	R:GTCTTCTTCCTTTGCTCGGAT	
HGF	F:TGCCATTCCAAATCGTCCTG	92
	R:ATTGTGGTGCCTTATACGTT	
MAPK10	F:ATGAGCCTCCATTTATTGTAC	148
	R:TTTGAGAACCGTAAACGTTG	

Note: F, forward primer; R, reverse primer.

## 3 Results

### 3.1 Raw data analysis and quality control

The original downstream sequences were filtered to obtain high-quality clean reads, and then the subsequent analysis was performed. As shown in Table 2, the sizes of the total clean reads of the four groups of samples were 94.02 G, 94.85 G, 93.66 G and 97.66 G. The Q30 ratios were all higher than 90%, indicating that the sequencing quality was reliable.

**TABLE 2:** Quality of data output.

Group	Raw reads	Clean	Clean	Q30/%
Group		Reads/G	Bases/G	
CK1	128,700,068	121,003,698	94.02	92.24
CK2	130,917,490	124,177,272	94.85	92.58
5i8d1	154,055,668	144,290,922	93.66	92.48
5i8d2	125,726,024	122,785,186	97.66	92.14

CK and 5i8d represent goat ear fibroblasts and reprogrammed mammary epithelial cells.

### 3.2 Sequence matching and splicing

The results of the quality-controlled clean reads compared to the goat reference genome were shown in Table 3. The results show that more than 96% of the clean reads in the control samples could locate sequenced sequences on the genome, 2.08%–2.17% had multiple alignment positions on the reference sequence, and the number of reads with unique alignment positions on the reference sequence accounted for more than 94% of the reads. The percentages of clean reads in the 5i8d samples that were compared to the genome were all greater than 95%, the percentage of reads that were compared to the reference sequence with multiple comparison positions was 2.23%–2.94%, and the percentage of reads with unique comparison positions was 92.80%–94.53%.

**TABLE 3:** Comparison of reads and reference genome.

Group	Total number of filtered reads	Number of reads that can be localized to the genome	Number of reads with multiple comparison positions on the reference sequence	Number of reads that have a unique comparison position on the reference sequence
Group	Total reads	Total mapped reads	Multiple mapped reads	Uniquely mapped reads
CK1	121,003,698	116,779,050 (96.51%)	2,631,155 (2.17%)	114,147,895 (94.34%)
CK2	124,177,272	120,090,486 (96.71%)	2,587,299 (2.08%)	117,503,187 (94.63%)
5i8d1	144,290,922	138,150,865 (95.74%)	4,248,247 (2.94%)	133,902,618 (92.80%)
5i8d2	122,785,186	118,813,067 (96.76%)	2,742,768 (2.23%)	116,070,299 (94.53%)

### 3.3 Identification of lncRNA transcripts

**FIGURE 1:**
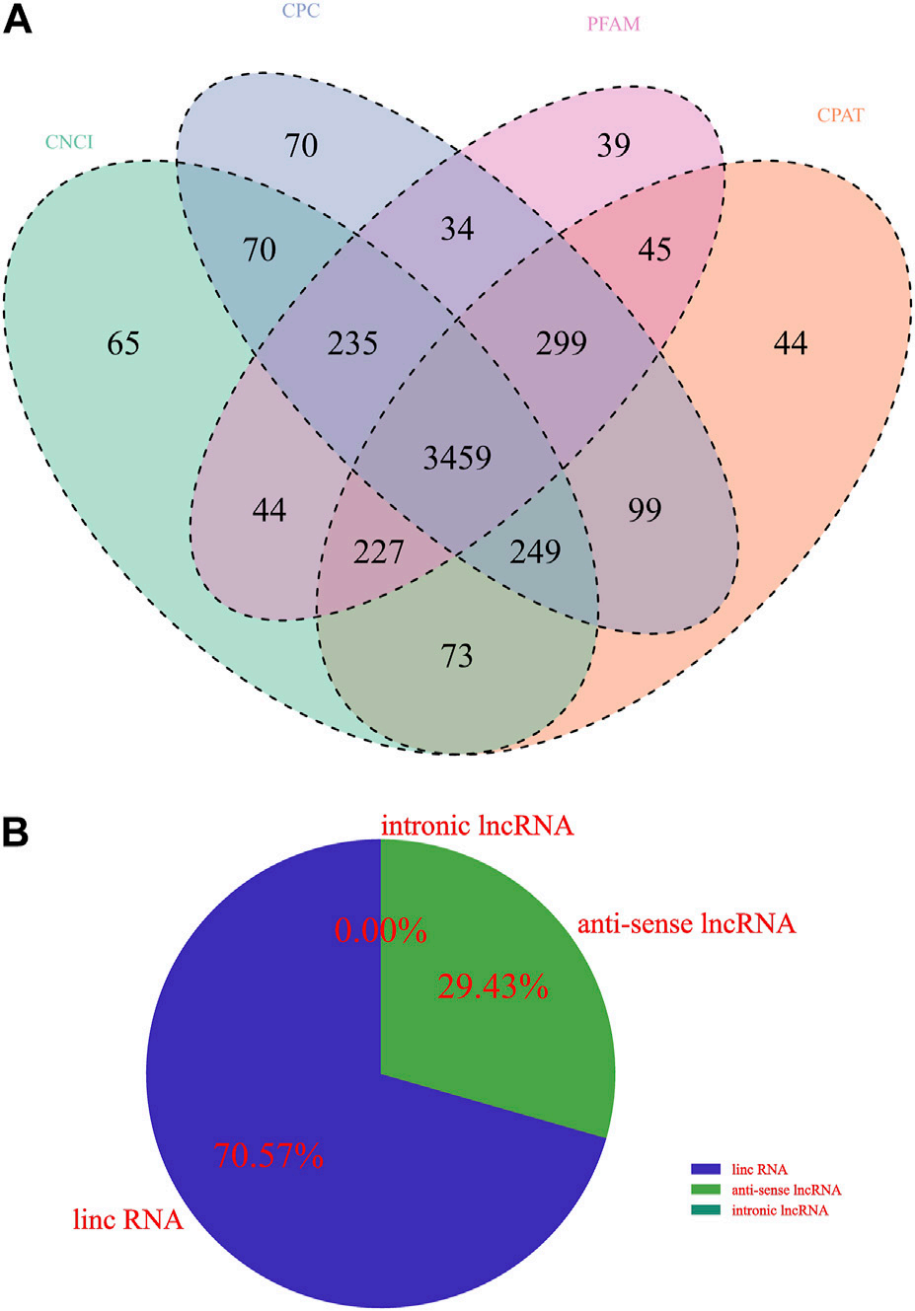
Identification of lncRNA transcripts. **(A)** Venn diagram showing different coding potential prediction methods to obtain subsequent lncRNA analysis data sets. **(B)** Identification of lncRNA transcripts.

Whether a new transcript has coding potential is the key condition to determine whether it is an lncRNA. The lncRNA information from the previous step was screened by combining various coding potential analysis software, mainly coding-noncoding index (CNCI) analysis, coding potential calculator (CPC) analysis, Pfam-scan protein structural domain analysis, and Coding Potential Assessment Tool (CPAT) analysis (animal only). The noncoding transcripts identified by the above coding potential prediction methods were counted, and the common and unique numbers of each method were displayed as Venn diagrams. The intersection of the predicted results was taken as the subsequent data set for novel lncRNA analysis (Figure 1A). Upon further classification of unknown lncRNAs, as shown in Figure 1B, lincRNAs accounted for 70.57%, intronic lncRNAs accounted for 29.43%, and no antisense lncRNAs were found.

### 3.4 Structure and characterization of lncRNAs

**FIGURE 2:**
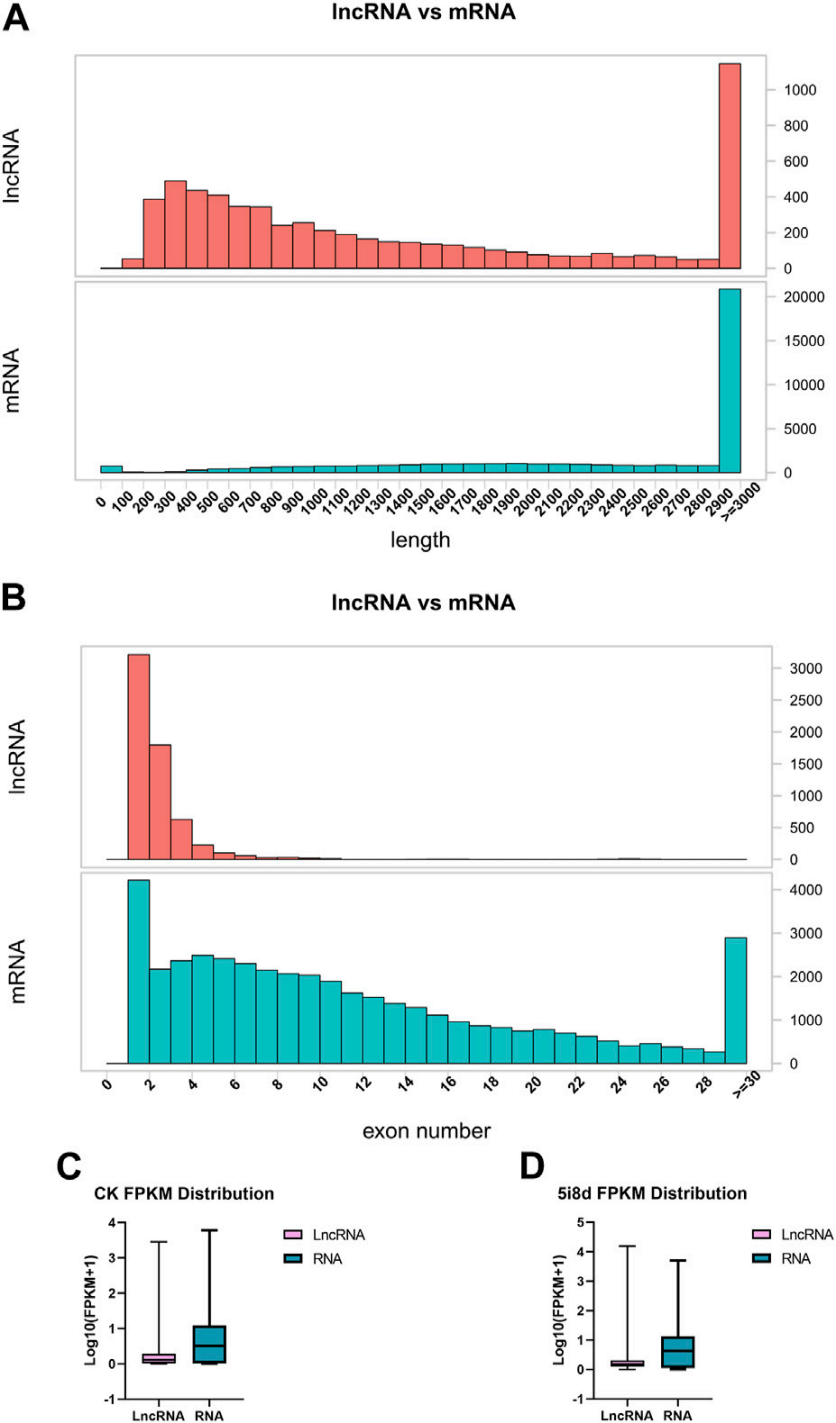
Comparative analysis of structural features of lncRNA and mRNA. **(A)** Comparison of lncRNA and mRNA length distribution. **(B)** Comparison of lncRNA and mRNA exon number. **(C)** Comparison of lncRNA and mRNA transcript expression in CK group. **(D)** Comparison of lncRNA and mRNA transcript expression in 5i8d group.

To investigate the basic characteristics of lncRNAs in fibroblasts and induced mammary epithelial cells of dairy goats in Guanzhong, the transcript length, exon number and transcript expression levels of lncRNAs and mRNAs were compared after bioinformatics analysis. The results showed that compared with mRNA, the lncRNA length interval was concentrated between 200–3,000 bp (Figure 2A), and the number of lncRNA exons was between 1 and 11, mostly between 1 and 3 (Figure 2B). The expression of lncRNAs obtained from 0 d to 8 d sequencing was compared with that of mRNAs, and the overall expression of mRNAs was found to be significantly higher than that of lncRNAs (Figures 2 C, D).

### 3.5 Differential expression analysis of lncRNAs

**FIGURE 3:**
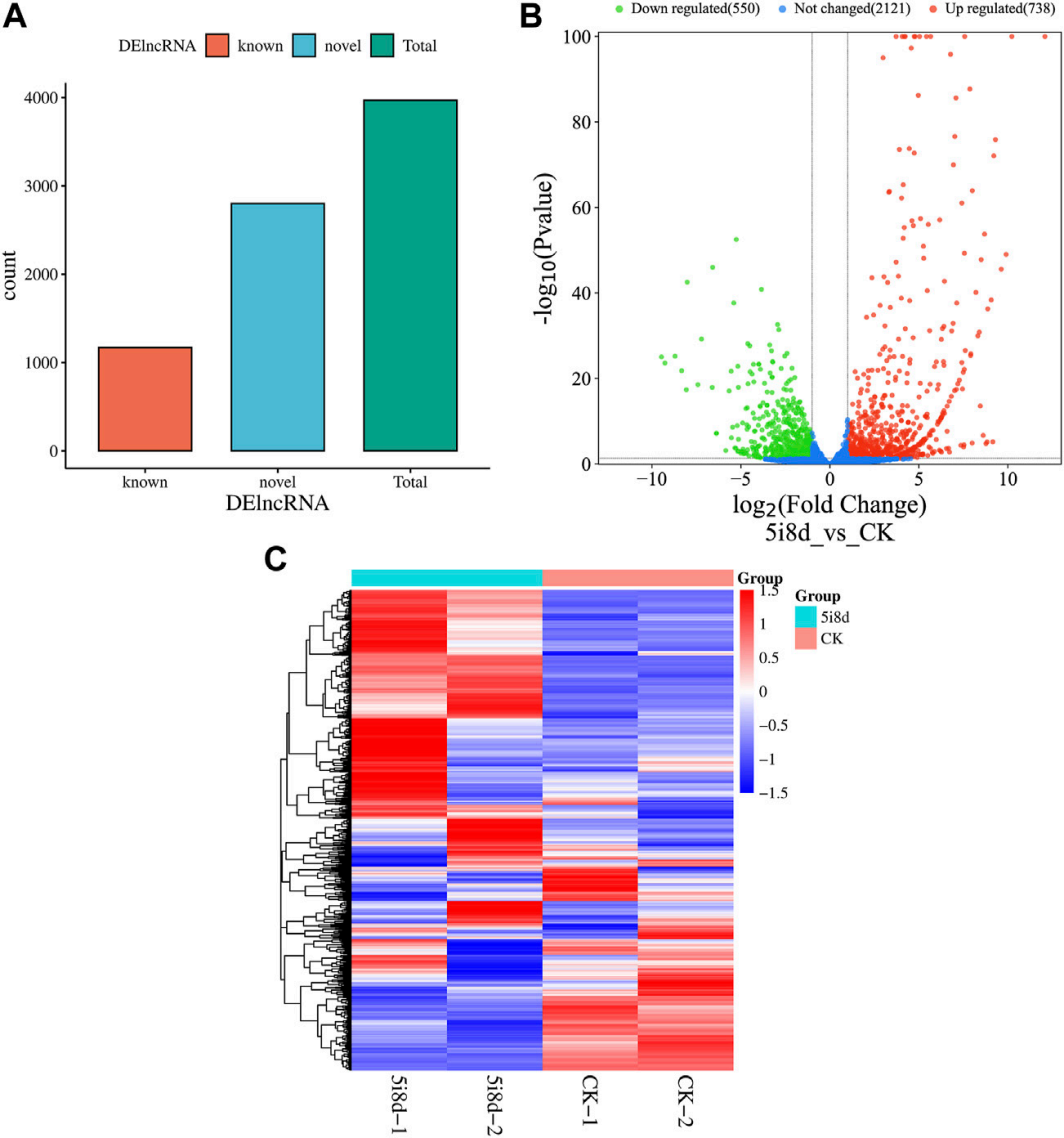
Differentially expressed lncRNA analysis. **(A)** Differentially expressed lncRNA expression type, green indicates total differentially expressed lncRNA number, red indicates known differentially expressed lncRNA number, blue indicates unknown differentially expressed lncRNA number. **(B)** Significant differentially expressed lncRNA volcano plot, green indicates downregulated, red upregulated expression. **(C)** Heatmap of differentially expressed (DE) lncRNAs.

DEseq was used for differential expression analysis, and genes with |log2Fold Change|≥2 and *p* < 0.05 were selected as significant differential expression screening conditions. The screening results were shown in Figure 3. The results showed that 3,970 candidate differential lncRNAs were obtained in the 5i8d group and the CK group, which included 1,170 known lncRNAs and 2,800 novel lncRNAs (Figure 3A). Further differential expression analysis of the goat fibroblast group and induced mammary epithelial cell group resulted in 1,288 significantly differentially expressed lncRNAs, of which 738 were upregulated and 550 were downregulated (Figure 3B). The heat maps of DE lncRNAs revealed different expression patterns between the 5i8d group and the CK group (Figure 3C).

### 3.6 Differentially expressed lncRNA-targeted mRNA prediction

**FIGURE 4:**
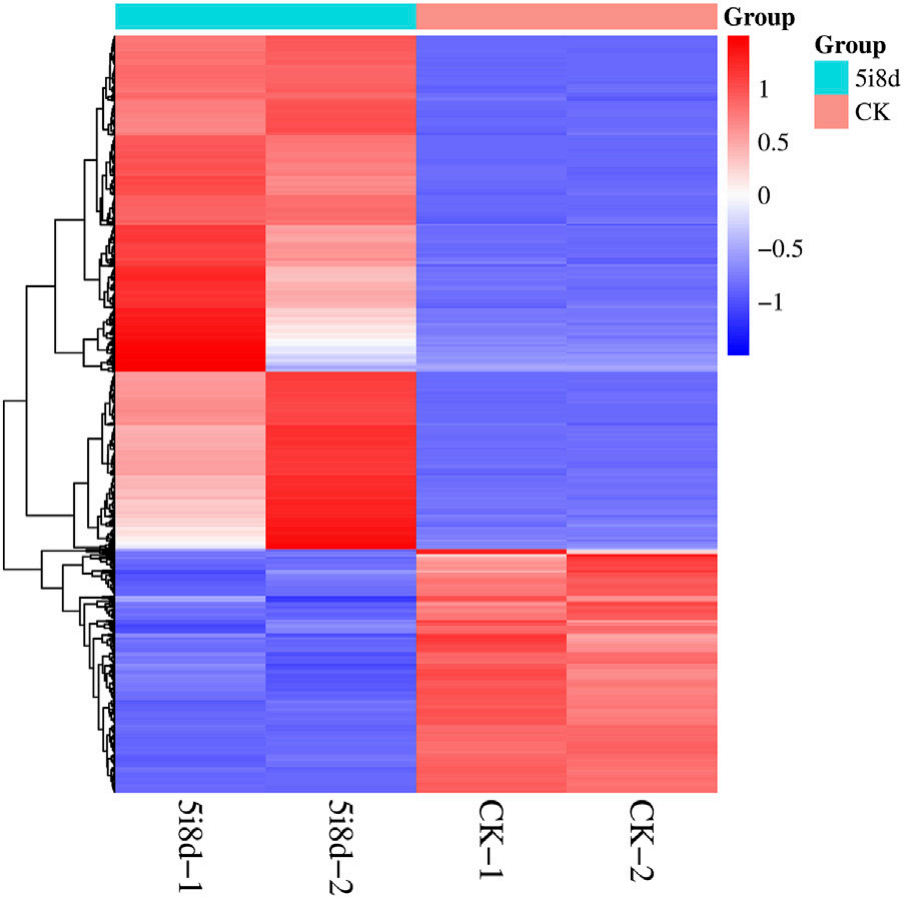
Heatmap of differentially expressed target genes.

To further investigate the role of DE lncRNAs in the reprogramming process of mammary epithelial cells, the possible target genes of DE lncRNAs were predicted by target analysis through cis and trans methods, and their functions were indirectly predicted through target genes. The target gene prediction results showed that there were 3,970 different lncRNAs, of which 2,800 were unknown and 1,170 were known, corresponding to 5,003 target genes. Among them, there were multiple significantly different lncRNAs with common target genes, such as MSTRG. 100417, MSTRG. 10063, and MSTRG. 10088 all correspond to the target gene MAPK10, and there were also lncRNAs with multiple variable shears targeting multiple coding genes, such as the coding genes FLT1, FGF9, HGF, and PDGFRA, which were potential targets of MSTRG.100336, and these lncRNAs exhibited regulatory effects in different directions. The heat maps of differently expressed target genes revealed different expression patterns between the 5i8d group and the CK group (Figure 4).

### 3.7 Validation of lncRNAs and target genes expression by RT-PCR

**FIGURE 5:**
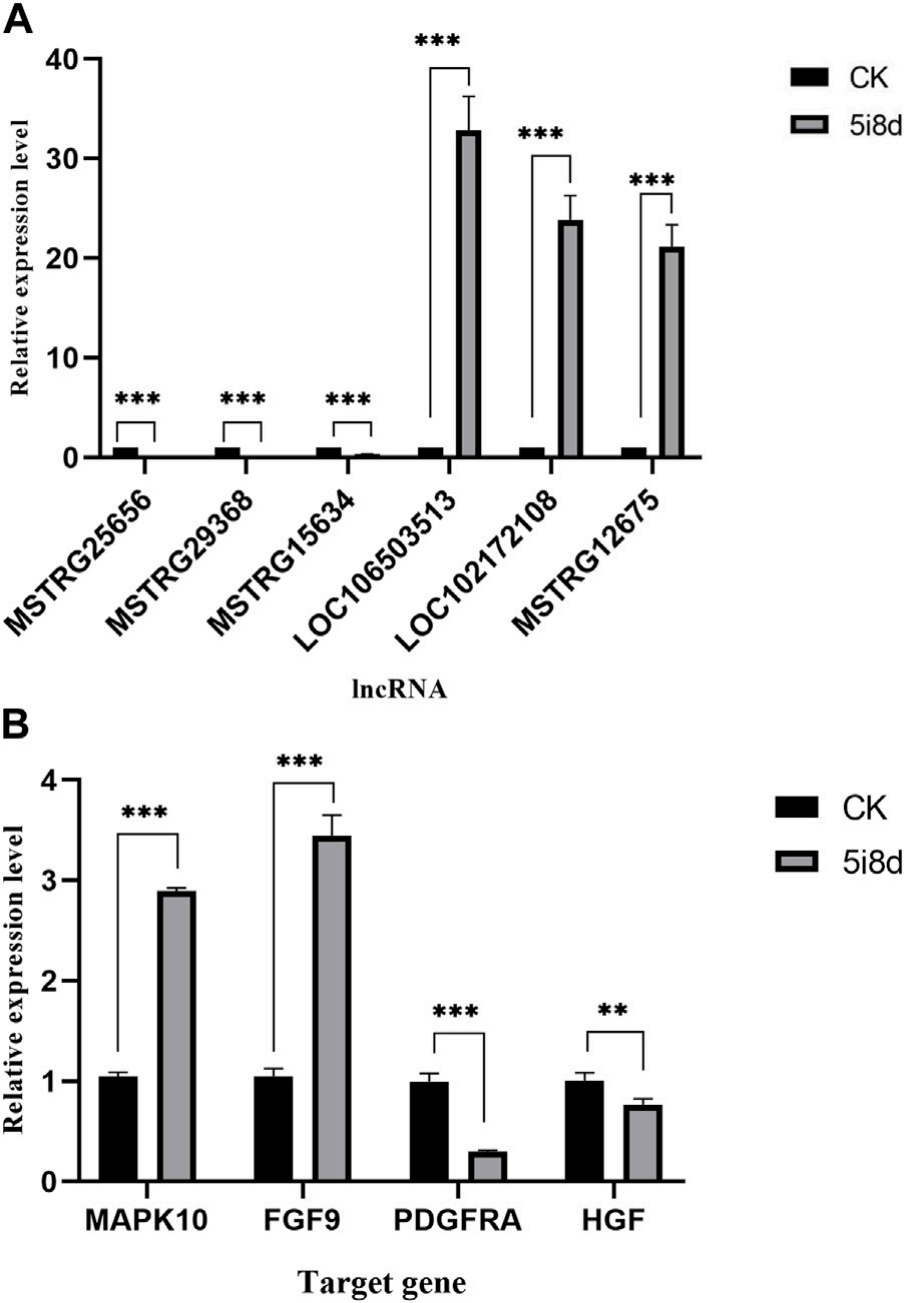
Comparison of the results of lncRNA-seq and RT-qPCR analysis of DE lncRNAs and target genes in CK group vs. 5i8d group. **(A)** Relative expression of DE lncRNAs expressed differently in different groups. **(B)** Relative expression of target genes expressed differently in different groups.

To further validate the sequencing results and detect their expression in induced goat mammary epithelial cells, six differentially expressed lncRNAs were randomly selected, in which MSTRG12675, LOC102172108, and LOC106503513 were upregulated in the transcriptome data, MSTRG25656, MSTRG29368, and MSTRG15634 were downregulated in the transcriptomic data Four candidate target genes were selected at the same time, among which MAPK10 and FGF9 were upregulated in transcriptome data, and PDGFRA and HGF were downregulated in transcriptome data. Its expression in goat fibroblasts and induced goat mammary epithelial cells was measured by RT-qPCR. As shown, the RT-qPCR results showed that the expression of MSTRG12675, LOC102172108, and LOC106503513 was upregulated and that of MSTRG25656, MSTRG29368, and MSTRG15634 was downregulated (Figure 5A), which were all consistent with the results of lncRNA-seq. The RT-qPCR results showed that the expression of MAPK10 and FGF9 was upregulated, and the expression of PDGFRA and HGF was downregulated (Figure 5B), which were all consistent with the sequencing results. This result demonstrated that the RT-qPCR results were consistent with the expression trend of the transcriptome sequencing results.

### 3.8 Functional annotation analysis of target genes: GOand KEGG

#### 3.8.1 GO enrichment analysis

**FIGURE 6:**
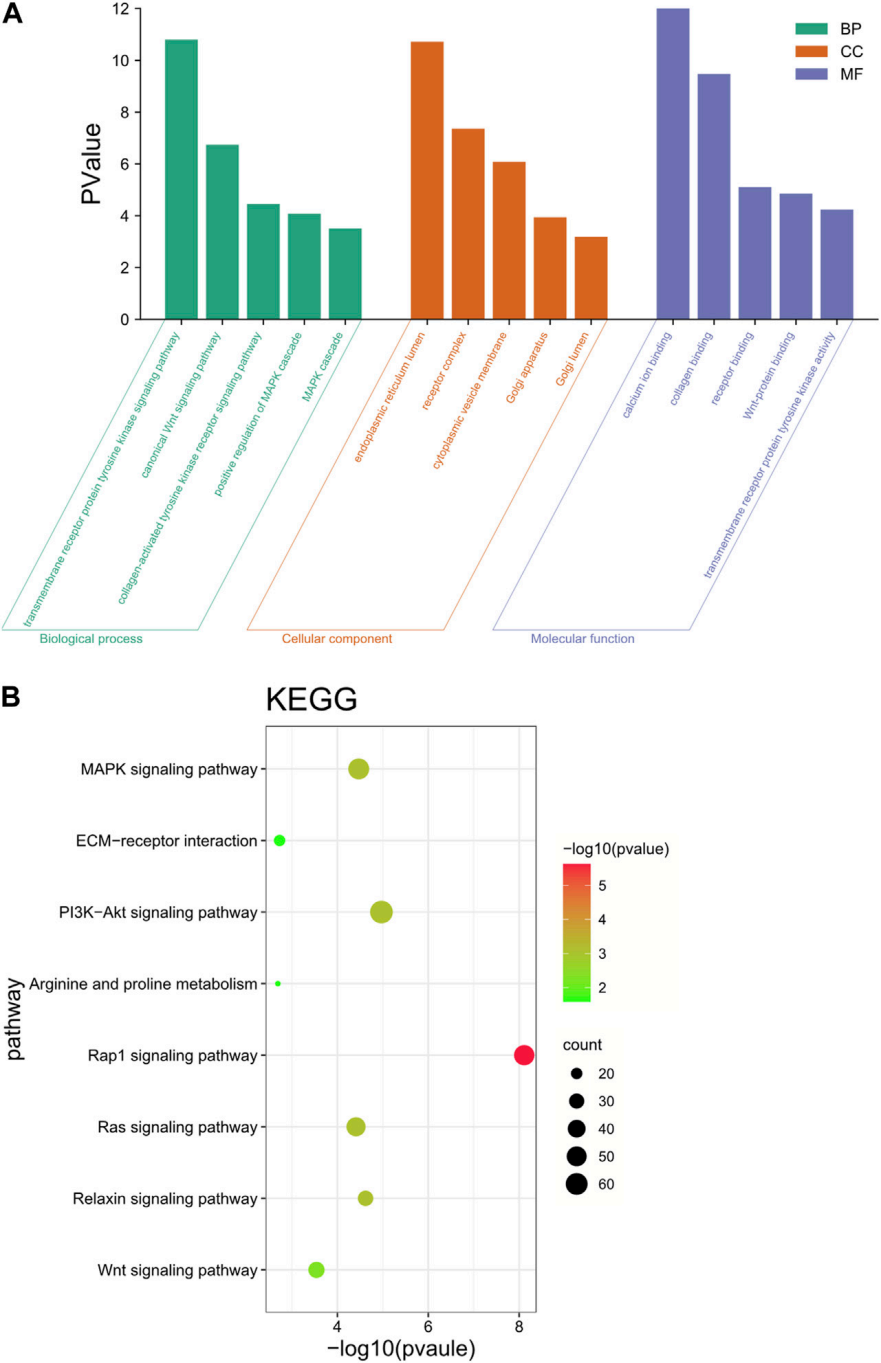
Functional annotation analysis of target genes. **(A)** GO enrichment analysis of differential lncRNA target genes. **(B)** KEGG enrichment analysis of differential lncRNA target genes.

These neighboring |log2Ratio|≥2 and *p* < 0.05 differentially significant target genes were analyzed for functional annotation, and the GO results were classified into three entries: biological process (BP), cellular component (CC) and molecular function (MF). The GO results were classified by three entries, namely, biological process (BP), cellular component (CC) and molecular function (MF). The results were shown in Figure 6A. These target genes were involved in the regulation of a variety of biological processes, including the regulation of metabolic processes, protein synthesis, receptor binding and regulation of tyrosine kinase activity.

#### 3.8.2 KEGG enrichment analysis

KEGG enrichment results showed that these target genes were mainly involved in the Wnt signaling pathway, PI3K-Akt signaling pathway, arginine and proline metabolism, ECM-receptor interaction, and MAPK signaling pathway (Figure 6B). These genes were significantly enriched in pathways that are associated with reprogramming and mammary gland development, while lncRNAs target these genes, thus suggesting that lncRNAs play a key role in reprogramming and mammary gland development.

### 3.9 Interaction network of lncRNA and mRNA

**FIGURE 7:**
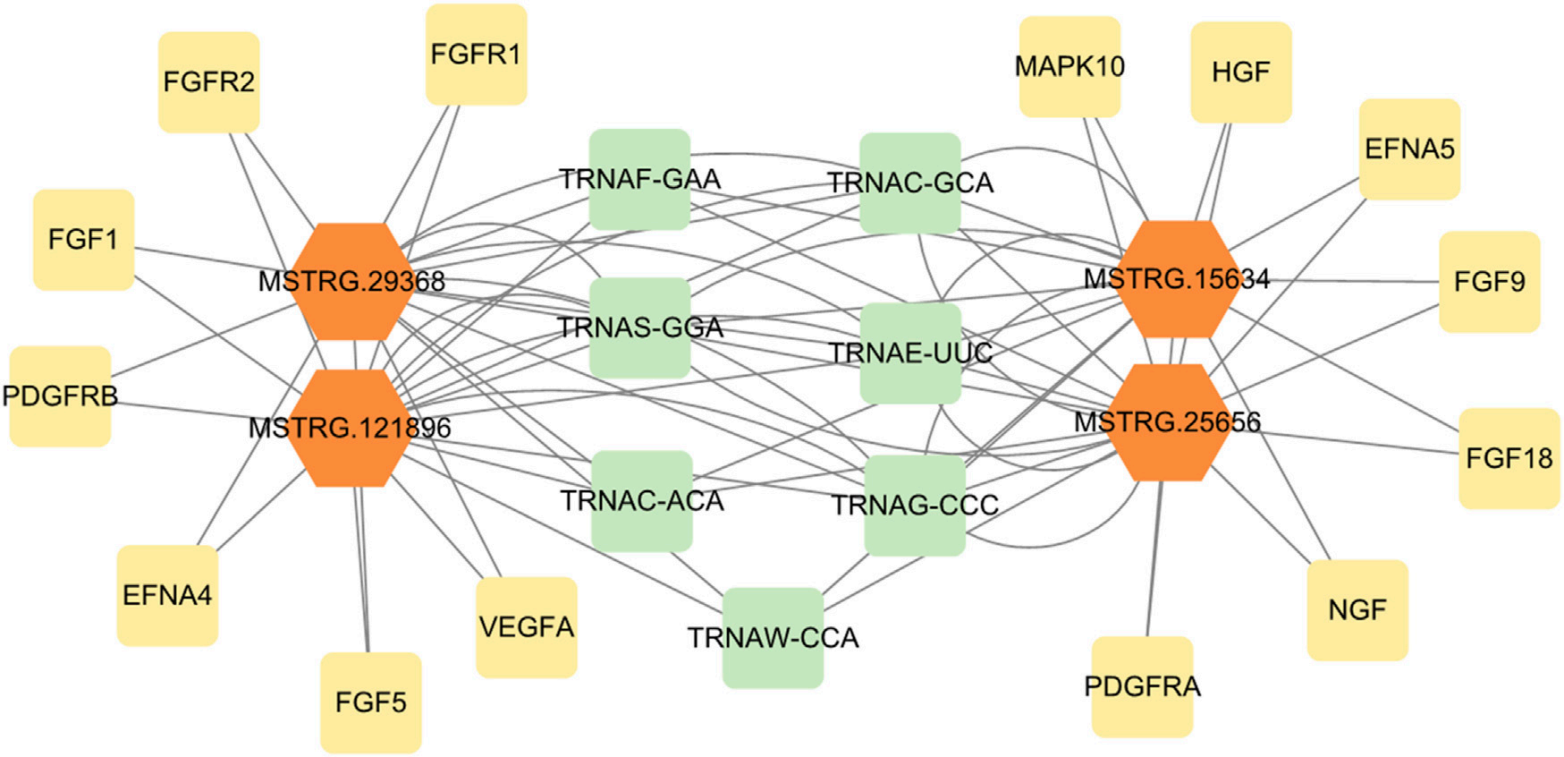
The regulatory network of differentially expressed lncRNAs and target genes.

The regulatory network analysis of differential lncRNAs with target genes was plotted based on the relationship of the identified differentially expressed lncRNA genes with mRNA genes and genes predicted by the cis and trans targets of lncRNAs (Figure 7). Yellow and green were target genes, and orange were lncRNAs. The results show that MSTRG.15634, MSTRG.25656, MSTRG.121896, and MSTRG.29368 were the differential lncRNAs that simultaneously target the most genes related to signaling pathways of mammary gland development and reprogramming, with MSTRG.15634 and MSTRG.25656 in MSTRG.15634 and MSTRG.25656 being the most upregulated differential lncRNAs that simultaneously targeted the above genes, and MSTRG.121896 and MSTRG.29368 being the most downregulated differential lncRNAs that targeted the above genes. The network plot results showed that MSTRG.15634 and MSTRG.25656 targeted MAPK10, FGF9, HGF, FGF18, EFNA5, PDGFRA, NGF, and MSTRG.121896 and MSTRG.29368 targeted FGF1, FGFR1, FGF5, FGFR2, PDGFRB, VEGFA, and EFNA4, while the above four lncRNAs targeted 7 transporter RNAs.

## 4 Discussion

lncRNAs play important roles in many biological activities, such as the dosage compensation effect, epigenetic regulation, cell cycle regulation, and cell differentiation regulation (Xiao et al., 2009), which has become a research hotspot in different scientific directions. In this study, transcriptome sequencing analysis of lncRNAs in Guanzhong dairy goat fibroblasts and induced mammary epithelial cells was performed, and then the target genes of lncRNAs were further predicted to reveal their functions. This study provides a theoretical basis for exploring the role of lncRNAs in reprogramming and lactation.

A total of 4,317 lncRNAs and 3,970 DElncRNAs were detected before and after the induction of the small molecule compounds. A total of 1,288 of these lncRNAs were significantly differentially expressed. In the present study, transcriptome sequencing analysis of lncRNAs in fibroblasts and induced mammary epithelial cells of dairy goats in Guanzhong was performed, and then the target genes of lncRNAs were further predicted to reveal their functions. The sequencing analysis results were consistent with those of (Li et al., 2019). These similarities suggest that the lncRNAs analyzed in this study were more reliable.

The expression level was verified by qRT‒PCR, and the trend was consistent with the lncRNA-Seq data. We enriched for differentially expressed lncRNA target genes, and GO enrichment revealed that the tyrosine kinase activity and WNT pathway involved in significantly enriched GO term entries were associated with mammary gland development and reprogramming; for example, tyrosine kinase influences mammary gland differentiation through prolactin receptors (Xie et al., 2002). Analysis and quantification of pluripotent pluripotency reprogramming showed that the WNT pathway undergoes extensive reprogramming and regulates various cellular and developmental processes (Hu et al., 2020). Therefore, these lncRNAs may play an important regulatory role in the process of mammary gland development.

KEGG enrichment significantly contains the MAPK signaling pathway, PI3K-Akt signaling pathway, Wnt signaling pathway, and relaxin signaling pathway related to mammary gland development; arginine and proline metabolism is a related pathway affecting milk protein synthesis by influencing the amino acid metabolism of milk proteins (Manni et al., 1998; Xie et al., 2002; HSU et al., 2005; Dinger et al., 2008; Xiao et al., 2009; Loewer et al., 2010; Guttman et al., 2011; Jardé and Dale, 2011; Meng et al., 2018; Li et al., 2019; Hu et al., 2020; Zhang et al., 2021; Sherman et al., 2022)^.^ There were also signaling pathways related to reprogramming ECM-receptor interactions, the Rap1 signaling pathway, the MAPK pathway, and the Ras signaling pathway (Mezl and Knox, 1977; Zwartkruis and Bos, 1999; Dickinson et al., 2010; Lopez-Vicente et al., 2011; Zhang et al., 2016; Xu et al., 2019; Dillon et al., 2021). Several genes exist in these pathways that were simultaneously involved in subprocesses, including MAPK10, FGF9, HGF, FGF18, EFNA5, PDGFRA, NGF, FGF1, FGFR1, FGF5, FGFR2, PDGFRB, VEGFA, and EFNA4. MAPK10 is a member of the MAPK signaling pathway, which is an important pathway for cell differentiation and proliferation. It has been shown that Fgf signaling affects the induction and development of the embryonic mammary gland by binding to a class of cell surface enzymes belonging to the family of receptor tyrosine kinases (FgfRs) (Dillon et al., 2004)^,^ that Fgf signaling has a role in normal mammary gland lobular alveolar development (Jackson et al., 1997a) and that Fgf signaling is also required for pregnancy-dependent lobular alveolar development in the mammary gland (Jackson et al., 1997b). HGF has mitogenic effects on human and mouse mammary epithelial cells, as well as kinetic and morphological effects, including the induction of extensive ductal branching and the formation of good lumens (Niranjan et al., 1995). HGF plays a key role in the estrogen-induced proliferation of mammary epithelial cells *in vivo* (Zhang et al., 2002). Efna5, a member of the ephrin gene family, has receptors whose spatiotemporal expression patterns are important for the morphogenesis and function of mammary epithelial tissue, and the Eph receptor acts as a signaling factor mediating hormones that are critical for the process of mammary gland expansion and regression (Perez White and Getsios, 2014). PDGF-BB expression upregulation affects mammary tissue fibrosis after *Staphylococcus aureus* infection, and PDGF-BB was found to be associated with breast fibrosis (Bi et al., 2020). Stromal PDGFRα signaling disrupts ECM homeostasis during mammary gland development, leading to increased mammary gland stiffness and increased tumor growth potential (Hammer et al., 2017). NGF and its high-affinity receptors are expressed during mammary gland development and lactation in sheep, and this expression is dependent on the stage of mammary gland development and lactation. NGF and its cognate receptors are expressed during mammary gland development, lactation, and degeneration in sheep through phosphatidylinositol 3-kinase (PI3K), and protein kinase B (AKT) plays a central role in promoting mammary cell survival during mammary gland development, lactation and degeneration (Colitti, 2015). Under the influence of hormonal and epithelial-stromal interactions, VEGF has multiple roles in the development and function of mammary glands, such as contributing to vascular development and supporting fat pads within the mammary gland (Hovey et al., 2001). Vascular endothelial growth factor affects mammary gland development by regulating mammary angiogenesis (Dangat et al., 2020).

In conclusion, all of the above target genes showed their role in reprogramming and mammary gland development relationships in previous studies, while the functional enrichment analysis of the present sequencing results showed that these target genes were also involved in reprogramming and mammary gland development-related pathways, among which two lncRNAs, MSTRG.15634 and MSTRG.25656, target both MAPK10, FGF9, HGF, MSTRG.25656, MSTRG.29368, and MSTRG.121896. The four lncRNAs involved in the above mentioned signaling pathways, through the regulation of their target genes, allowed fibroblasts to undergo fate transformation and obtain functional mammary epithelial cells.

## 5 Conclusion

We used lncRNA-Seq data of the transdifferentiation process to understand the expression profile of lncRNAs in the reprogramming of Guanzhong dairy goat fibroblasts into mammary epithelial cells, while analysis of the data revealed that four lncRNAs, MSTRG.15634, MSTRG.25656, MSTRG.29368, and MSTRG.121896, may have a key role in mammary gland development and lactation. Our findings can provide new ideas for further study of lncRNAs and mRNAs associated with mammary gland development and reprogramming and provide a theoretical basis for further elaboration of the reprogramming process from dairy goat fibroblasts to mammary epithelial cells in Guanzhong and the molecular mechanism of mammary gland development.

## Data Availability

The datasets presented in this study can be found in online repositories. The names of the repository/repositories and accession number(s) can be found below: https://www.ncbi.nlm.nih.gov/geo/; GSE142551.
